# A Review of Effects of Pandemic on the Patients of Obsessive-Compulsive Disorder

**DOI:** 10.7759/cureus.30628

**Published:** 2022-10-24

**Authors:** Mihika V Gokhale, Swarupa Chakole

**Affiliations:** 1 Community Medicine, Jawaharlal Nehru Medical College, Datta Meghe Institute of Medical Sciences, Wardha, IND

**Keywords:** exposure and response prevention, covid-19, swine flu, obsessive compulsive disorder, pandemic

## Abstract

In today's world, physical as well as mental health both play a crucial role. However, various pandemics have had adverse effects not only on the physical but mental and social health too. With the various preventive measures introduced to handle various pandemics, it becomes more critical to understand how the preventive measures affect the lives of patients suffering from Obsessive-Compulsive Disorder (OCD). The fear of getting contaminated and the fear of being affected by the disease are the characteristics that are already present in the patients with OCD. The various preventive measures introduced during pandemics may exacerbate the symptoms causing major discomfort to the patients. Due to the stigma present around the various mental health issues, it becomes difficult for early detection and prompt treatment. The failure to receive the treatment may adversely affect the patient's life, and it may also decrease their social interactions. In case of OCD, the already existing symptoms of OCD may even deteriorate, so effective management becomes a requisite. Cognitive Behavioural Therapy (CBT), along with Exposure and Response Prevention (ERP), has shown effective improvement in those with OCD who are exposed to various risks. The compulsions performed are lessened or even prevented. But in the time of pandemics, where preventive measures are essential not only to prevent being affected by the disease, but also to prevent its outbreak, Exposure and Response Prevention therapy should also be analyzed.

## Introduction and background

Health is not only about being physically healthy but being socially and mentally healthy as well. Most of the time, mental health is often neglected due to either lack of awareness or stigmatization of certain diseases. Hence many mental health conditions go under-reported. When these psychiatric conditions are not diagnosed and treated early, the disease may progress to the chronic stage and become extremely uncomfortable for patients with psychiatric conditions. One such condition is Obsessive-Compulsive Disorder (OCD). Obsessive-Compulsive Disorder is a condition that consists of some thoughts that are intrusive and bring discomfort to the patients. Patients with OCD may use several rituals or compulsions to decrease stress and anxiety levels. These compulsions do not give pleasure, but they only help reduce stress for a limited time. OCD usually goes under-reported and is under-treated. The possible reasons may be the embarrassment faced by the patients on account of the thoughts involving unpleasant sexual images, ideas about inappropriate behavior in public places, etc. Intervention is needed at an early stage, as the delay may cause severe debilitation. The World Health Organization has named OCD among the 10 disabling disorders. Patients who have OCD often avoid uncomfortable situations, and this may lead to a decreased life quality and also, reduced social interactions [1]. There is an intense fear of contamination and also a fear of acquiring certain diseases. This affects a person's capability to reason, and hence, it intervenes in the day-to-day lifestyle. Hence, the emergence of several contagious diseases may increase or aggravate the symptoms of a patient dealing with OCD.

In the past few decades, the world has witnessed the emergence of new diseases in various geographical regions. A strange kind of pneumonia was reported in March 2003, which was termed Severe Acute Respiratory Syndrome (SARS) by the World Health Organization [2]. The world was alarmed and shocked by the swift increase in the number of cases of Severe Acute Respiratory Syndrome in 2003 across the continents, with a mortality rate of 10%, and its detrimental effects on the economies of various regions [3]. Studies have shown a reduced risk of disease transmission by separating people physically from healthcare workers, visitors to the hospital, and patients suffering from the disease. Handwashing, although an effective measure was recognized as having poor compliance among healthcare workers. Several other measures, including environmental disinfection, education, and training, were also employed for the prevention of the disease. But along with physical health, it also started affecting mental health. The healthcare workers also observed several psychological impacts. The fear of transmission of the disease, financial burden, and lifestyle changes was a part of this. Stress and anxiety were also reported in healthcare workers [4]. In 2009, the world witnessed another emerging disease, the swine flu, which soon became a pandemic in June 2009 [5]. Direct contact and droplet inhalation became the routes for the human to human transmission [6]. A disproportionate disease was caused by the virus H1N1, similar to seasonal influenza [7]. Handwashing and maintaining a safe distance from people showing flu-like symptoms were considered the best practices to prevent the transmission of the disease [8]. Since the fear of acquiring the infection may have an adverse effect on a person's day-to-day lifestyle, there were several studies conducted to study the relationship between worries about the disease and anxiety levels. Recently, the Coronavirus Disease 2019 (COVID-19), with its catastrophic effects, resulted in more than six million deaths worldwide as of march 2022 [9]. This has resulted in the emergence of one of the most critical global health crises. In December 2019, an outbreak was reported of pneumonia of an unknown cause in Wuhan. After analyzing the condition worldwide, COVID-19 was announced as a pandemic by the World Health Organization [10]. It has not only overwhelmed several healthcare systems across the globe but has also impacted people's livelihoods due to shutdowns which ultimately resulted in a negative impact on the global economy [9]. It is highly contagious and is transmitted through both direct and indirect contact. Washing hands for a minimum of 20 seconds, maintaining a safe distance of 2 meters, avoiding shaking hands while greeting, and maintaining a safe distance from those who show symptoms of the disease are among the few preventive measures taken to reduce transmission [11]. But this has affected the mental health of people in general. New symptoms may appear, and the already existing symptoms of various psychiatric illnesses may exacerbate during various outbreaks. Conditions like depression, anxiety, post-traumatic stress disorder (PTSD), panic attack, and suicidal ideations are certain psychiatric conditions that have been associated with pandemics. And hence these conditions have been felt by the general population as well during the COVID-19 pandemic [12]. COVID-19 has also produced a significant challenge regarding its neurological consequences. These may include olfactory and gustatory disorders and headaches and may also affect cognition [13]. COVID-19 has also impacted several social issues. These include gambling and alcoholism. The pandemic diversely impacted gambling. It observed a decline in the problems of the present and the future but increased problematic gambling [14]. Substance use disorder, especially alcohol use disorder, has also impacted people’s life. There has been a bidirectional relationship that has been observed, where stress increases alcohol consumption, and this, in turn, produces more stress. In some countries, it was also observed that people who were heavy drinkers experienced withdrawal symptoms as they could not access it [15]. With the emergence of new diseases, not only is an individual's physical health affected, but mental health has also witnessed an adverse effect. Since the fear of contamination is an essential feature of OCD, it becomes crucial to identify the effects of preventive measures on OCD patients. Identification of these effects would be helpful in effectively managing patients dealing with OCD. And this would probably help deal with the patients in future pandemics. A focused literature search was conducted with the help of various databases that included PubMed and Google Scholar. Several articles were short-listed. Search terms were mainly based on epidemiology (e.g., "Obsessive Compulsive Disorder," "pandemic") impacts of contagious diseases on Obsessive Compulsive Disorder and behavioral aspects. Full texts of the articles were reviewed, and references were listed.

## Review

**Figure 1:**
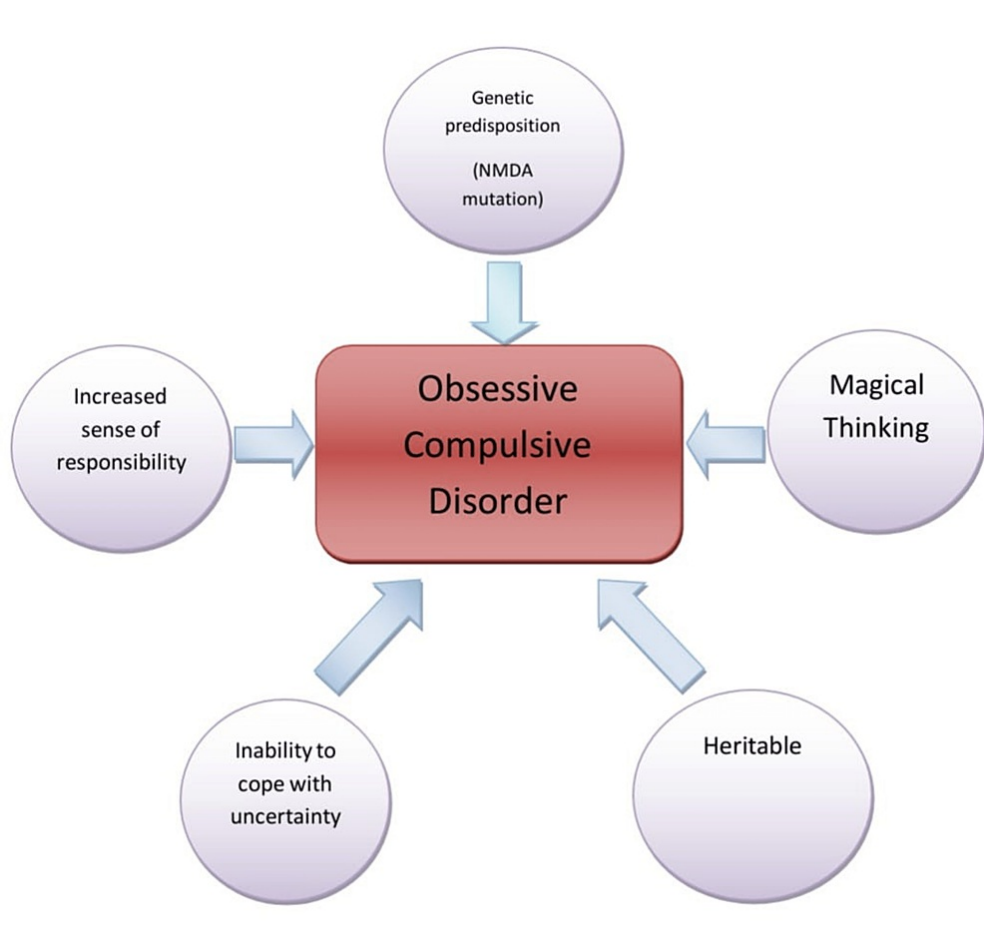
Predisposing risk factors [1] Figure created by the author from the information in this source

Pandemics have consistently shown the world the worst phases, where several deaths and high transmission rates of these diseases have been detrimental to the general population. These have not only resulted in a physical disaster, but their adverse impacts on the mental and social well-being of the people are also essential consequences. The fear of losing loved ones or being affected by the devastating effect of the pandemic has affected people psychologically. Hence, because of all these factors, stress and anxiety levels have increased during the times of pandemic. Especially patients with OCD may experience incredible difficulty because of their fears. Usually, in 50% of the patients who are suffering from OCD, the symptoms start appearing in childhood and adolescence. Symptoms appearing over the age of 40 are unusual. It is also found that there is a presence of a coexisting psychiatric diagnosis, usually anxiety disorders, in about 90% of the people dealing with OCD. There is almost two times more probability for a postpartum female to develop OCD than a female from the general population. But most of the time, these cases go under-reported. The effective management of OCD is often refused by the patients, aborted by the patients, or fails to respond in about 30% of the cases of OCD [1]. There are various predisposing factors attributing to the development of OCD. The predisposing risk factors of OCD are mentioned in Figure 1.

SARS outbreak

During the Severe Acute Respiratory Syndrome outbreak, the physical and mental health of people was affected. According to a study, Severe Acute Respiratory Syndrome outbreak-related negative information led to increased risk perception and irrational fear [16]. According to another study, depression and symptoms of post-traumatic stress disorder were present in those who were quarantined [17]. In 2003, with the use of traditional public health intervention, it became possible to contain Severe Acute Respiratory Syndrome among the human population [18]. But these preventive measures exacerbated certain psychiatric conditions.

Swine flu

During the Swine Flu pandemic in the year 2009, many opportunities emerged to examine the association between health anxiety and fears of the pandemic. The most common symptom in patients dealing with OCD is the fear of contamination, and this urges them to take extra preventive measures to avoid acquiring the disease [5]. There are chances where patients with OCD seek reassurance and may undergo several medical examinations that may give them temporary relief, but this may be only for a short term [19]. Excess study of information regarding diseases and overestimating the symptoms are some of the major characteristics of OCD patients. Studies have shown that pre-existing psychiatric disorders are exacerbated in high-risk populations.

COVID-19

The most recent outbreak of the Coronavirus disease has brought about a sea change in the day-to-day lifestyle. The preventive measures, including the shutdowns and social distancing, have affected people not only socially but also mentally. Months of lockdown have been detrimental to the global economy. Restricted transportation and almost no social interactions have had adverse impacts on the day-to-day lifestyle. After December 2019, an exponential increase in cases was seen. The first case reported outside China was on January 11 in Thailand, and within a few months, except in Antarctica, it spread to various continents. India reported its first-ever COVID-19 case in January 2020. In 2020, on March 12, India reported its first COVID-19-related death. The disease spread across all the states of India by the second week of April, except Sikkim [10]. Individuals of all ages are susceptible to developing the disease, but people who are above 60 years of age and have specific comorbidities are at a greater risk of developing the infection [9]. OCD patients usually have an urge to use excessive preventive techniques to avoid getting infected. Studies have shown that in the trying times of the COVID-19 pandemic worsening in the conditions of patients dealing with OCD was observed. A new obsession with developing COVID-19 symptoms has also been reported in studies. Suicidal thoughts have also been observed in OCD patients than in the normal population [20]. During the COVID-19 pandemic, the preventive measures that were suggested were similar to the characteristics of those with OCD, like handwashing and isolation, and this exacerbated the pre-existing symptoms. Certain studies had shown that OCD patients before the pandemic started experiencing increased levels of severity in the symptoms related to OCD during the pandemic, which was mainly due to the stress that was induced because of the pandemic. It was also observed that not only the people at risk had developed psychological issues, but people from the general population also developed some psychological disturbances apart from the symptoms of OCD. The symptoms that were observed in the OCD patients were isolating themselves, excessive handwashing avoiding some foodstuff, and also excessive disinfection. Although there is finite information regarding the effects of excessive handwashing on physical health during the pandemic, studies that were conducted before the pandemic stated that excessive handwashing might lead to the development of dermatological problems [21]. Even healthy people may often develop mental health issues due to the quarantine-related continuous isolation from social life. Since the advisories issued mainly focused on washing hands, symptoms related to OCD may worsen [22]. Effective management strategies were adopted, but preventive medicine like vaccination was considered effective along with a long-term preventive measure. Despite the evidence shown by various studies about the safety and efficacy of the vaccines used in COVID-19, vaccine hesitancy poses a significant challenge. Various myths like the vaccine may infect one with COVID-19, or it may lead to infertility issues, may increase anxiety levels, thus resulting in vaccine hesitancy. This is especially high in patients with certain mental health conditions. Lack of motivation may lead to vaccine hesitancy in patients suffering from depressive disorders. Fear of getting injected with germs can also lead to vaccine hesitancy in those suffering from OCD. These may increase the already existing anxiety about the vaccine [23]. Any stress-inducing agent would worsen the psychiatric symptoms, and the pandemic acted as a stress-inducing agent. It not only elevated the anxiety levels but also a disproportionate response to the intrusive thoughts [24]. It has been observed that during the pandemic, people spent most of the time gathering information and news related to the pandemic through media which mainly emphasizes the importance of personal hygiene, resulting in the worsening of the symptoms of OCD patients [25]. The various preventive measures that were adopted in dealing with the outbreaks are enumerated in Table 1 below.

**Table 1:** Various preventive measures adopted for various outbreaks [4,8,11]

Outbreak	Preventive Measures
2003 SARS	Hand washing, Isolation, Physical Spacing, Environmental Decontamination
2009 Swine Flu	Hand washing, Vaccination, Quarantine, Wearing mask
2019 COVID	Isolation, Hand washing, Mask, Disinfectants

Now, it becomes essential for the clinician to obtain specific treatment modalities for the treatment of patients who are already suffering from the fear of contamination. Preventing handwashing while being exposed to the fear of getting the infection is primarily an essential part. But since, during a pandemic, how much handwashing is needed is still a tricky question [26]. Exposure and Response Prevention has been identified as one of the effective ways to manage OCD, and there have been many patients who have experienced a lot of improvement in their condition [27]. Habituating an individual with fear by exposing the patient to that fear leads to the achievement of extinction of the severe symptoms of OCD [40]. Studies have shown that Cognitive Behavioural Therapy and Exposure and Response Prevention effectively reduce symptoms of OCD [28]. It is essential that OCD is detected early and appropriate treatment methodology is started. It has also been observed that family members participate in the compulsions that are performed by the patient. This may reduce the anxiety for a limited time, but it may exacerbate it in the future when exposed to a similar fear. For the treatment planning, it is also crucial to understand how much the patient's family is aware of OCD, family accommodation, and the family history of other psychiatric disorders also plays an important role. With reduced participation by the family members in the compulsions performed by the patients of OCD, effective treatment has been witnessed [29]. With the upcoming challenges of several diseases, it becomes crucial to understand and deal with psychological issues before the condition worsens. There is still a problem with mental disorders because they are underestimated due to the lack of acknowledgment of a relationship between mental illness and other physical illnesses [30]. Studies have shown that people's attitude and behavior toward patients who have a particular mental illness is negative [31]. It is crucial to be educated about the symptoms associated with various mental disorders to assist patients suffering from certain mental illnesses and provide them with the treatment they require [32]. Several treatments are effective and are different for various disorders [33]. Several patients globally suffer from mental health issues but do not receive treatment associated with mental disorders [34]. There is a burden on the families, individuals, and society that any mental health issue would create [35]. For the last three decades, psychological disorders and various psychiatric diseases have risen [36]. Hence it becomes essential for clinicians to use evidence-based explanatory models for the people they serve [37]. Mental health issues are still associated with social stigmas [38]. Critical interventions are essential as, in the absence, the prevalence rates may increase along with the associated cost [39].

## Conclusions

The 21^st^ century has observed pandemics in their worst forms, accounting for the deaths of millions across the globe. The aftereffects of these pandemics are debilitating and have had a detrimental effect on people's lifestyles. The various governments and health authorities recommended preventive measures that have helped contain the disease and prevent its further transmission. Still, the effects they have created in people's minds are challenging to handle. Lockdown for months has reduced the quality of life because of decreased social interactions. The financial loss of the people has adversely affected their lifestyles and the global economy. Living with the fear of pandemics has also led to several mental health-related problems. OCD patients, who are already under the suspicion of contamination and acquiring various diseases, may experience increased severity in their already existing condition. Frequent and excessive use of preventive measures to avoid contamination exacerbates the situation. Lack of these preventive measures lead to an increase in anxiety levels. The patient may have a strong urge to use them. And in the absence, distress may occur. The fear of getting infected increases, and several thoughts regarding the devastating effects of the disease start developing. But if these preventive measures are performed, there is a temporary relief from anxiety, but it may trigger in future when exposed to the same fear. This leads to the formation of a vicious cycle. Exposure and Response Prevention is based on breaking this cycle. Anxiety increases to a peak when these preventive measures lack, but gradually decreases by regular practice of Exposure and Response Prevention therapy. Hence, it becomes essential to study the ill effects of the pandemic not only on the physical but mental health-related matters, as this may give clinicians better and more effective strategies for patients with OCD, or else the prevalence rates of the diseases may continue to rise.
